# Effects of placental cord drainage in the third stage of labour: A meta-analysis

**DOI:** 10.1038/s41598-017-07722-7

**Published:** 2017-08-01

**Authors:** Hang-lin Wu, Xiao-wen Chen, Pei Wang, Qiu-meng Wang

**Affiliations:** Department of Obstetrics and Gynaecology, Hangzhou Women’s Hospital, Hangzhou, Zhejiang China

## Abstract

Observational studies have demonstrated that placental cord drainage can shorten the length of the third stage of labour and reduce blood loss during vaginal deliveries. The aim of our work was to evaluate the existing evidence for the effectiveness of placental cord drainage in the third stage of labour. PubMed, Embase, the Cochrane Library, Web of Science, Google Scholar and 50 journals were searched up to the 4th of June, 2017. Randomized controlled trials comparing placental cord drainage with no cord drainage in the third stage of labour during vaginal delivery were included. Nine studies with 2653 participants were included. Compared with clamping the umbilical cord, umbilical cord drainage during the third stage of labour shortened the third-stage duration by 2.28 minutes (95% confidence interval (CI), −3.22 to −1.33), but did not reduce the amount of blood loss (−31.99 mL, −86.08 to 22.09). For women with normal vaginal deliveries, the incidence of postpartum haemorrhage was reduced by 3%. Placental cord drainage is a simple and non-invasive procedure that should be considered after delayed cord clamping. Further studies about the physiological processes and effects of placental cord drainage in additional circumstances are needed.

## Introduction

The third stage of labour is defined as the period from the birth of the baby to the expulsion of the placenta. Management of the third stage is related to the obstetric complications and maternal prognosis. Maternal haemorrhage and maternal hypertensive disorders were the dominant causes of maternal death in 2015 and together accounted for more than 50% of all maternal deaths^1^. Prolongation of the third stage of labour leads to an increased complication rate, particularly the incidence of postpartum haemorrhage (PPH)^2^. Active management and expectant management are regarded as two different approaches to the clinical management of the third stage of labour. Active management of the third stage involves oxytocic drugs or early cord clamping (especially before cord pulsation ceases) or controlled cord traction (CCT); the latter involves the process of waiting for the spontaneous separation and expulsion of the placenta without artificial intervention but can be aided by gravity or nipple stimulation.

As a common method used to prevent PPH, umbilical vein oxytocin injection can result in earlier uterine contraction and thus accelerate the process of placental separation^3^. It has been suggested that prophylactic oxytocin use in labour decreases the incidence of PPH^4^. CCT is defined as pulling on the cord while applying counter pressure to aid the expulsion of the placenta. Recently, a Cochrane review about the effects of CCT found that when managed with CCT, the incidence of PPH and the duration of the third stage of labour were reduced. However, CCT may make mothers uncomfortable, and specific training is needed for midwives to perform CCT^5^.

Recently, different types of active management have been suggested to prevent PPH; for example, prophylactic ergometrine-oxytocin during the third stage of labour^6^, prostaglandins^7^ and oxytocin agonists used during the third stage of labour^8^. However, these methods are used by a minority of midwives and still require further evaluation.

Placental cord drainage (PCD) is defined as the unclamping the maternal side of the umbilical cord, thereby permitting the blood from the placenta to drain freely into a vessel immediately after the clamping and cutting of the umbilical cord. In 1999, Razmkhah first reported that the duration of the third stage of labour could be shortened via PCD^9^. Subsequently, some studies reported similar results^10–12^, but one study reported no benefit of this method^13^. A Cochrane review in 2011 concluded that the implementation of PCD resulted in 77-mL reductions in blood loss and 3-min reductions in the duration of the third stage of labour^14^.

Recently, a few studies have reported on the effects of PCD in the management of the third stage of labour. Asicioglu reported that active management with PCD during the third stage of labour significantly reduced postpartum blood loss and third-stage duration^15^. Roy found that PCD was effective in reducing the blood loss and third-stage duration^16^. However, Lankeshwara reported opposite results by pointing out that PCD as a part of the management of the third stage of labour increased blood loss, incidence of PPH and the duration of the third stage^17^. In 2015 Amorim concluded that PCD had no effect on reducing either the duration of the third stage of labour or blood loss^18^.

The aim of our work was to evaluate the existing evidence for the effectiveness of placental cord drainage during the third stage of labour following vaginal delivery.

## Methods

### Search strategy and selection criteria

The meta-analysis was performed and reported in accordance with the recommended methods using the PRISMA checklist (Supplementary Appendix S1). A comprehensive search was performed to collect all of the studies about PCD, from which we selected the randomized controlled trials for evaluation. We searched five electronic databases for articles published up to 4th of June, 2017, including PubMed, Embase, the Cochrane Library, Web of Science and Google Scholar. Additionally, we also searched 50 related journals, the majority of which are currently on the initiative lists of Core Outcomes in Women’s and Newborn Health. No language or publication type limits were applied. The reference lists of the selected articles and reviews were hand searched to identify any other relevant articles. The medical subject headings (Mesh) terms used were the following: ‘drainage’, ‘postpartum haemorrhage’, ‘placenta, retained’ and ‘labour stage, third’. Details about the search strategy can be found in the Supplementary Appendix S2.

The studies selected were required to include a comparison between PCD and no cord drainage after the clamping and cutting of the umbilical cord during the third stage of labour following vaginal delivery. Studies in which interventions such as a prophylactic uterotonic or CCT were performed with PCD were also included. If the interventions of these studies were performed differently in the experimental and control groups, the study was excluded. Quasi-randomized studies were also excluded. If we encountered difficulty evaluating the quality of the study, we attempted to contact the author for more information.

The primary outcomes of interest were the following: the duration of the third stage of labour, postpartum blood loss, the incidence of postpartum haemorrhage, the incidence of a retained placenta, the requirement for the manual removal of the placenta, the need for blood transfusion, changes in maternal haemoglobin after delivery, additional uterotonic drugs required, and adverse events at the time of drainage.

### Data extraction and quality assessment

Two independent researchers (H-L.W. and P.W.) performed separate searches. Data extraction and accuracy verification were resolved by other researchers (X-W.C. and Q-M.W.). Duplicate or irrelevant articles were excluded based on screenings of the titles and abstracts. The full texts of all remaining articles were screened.

All researchers assessed the risks of bias of the studies independently according to the Cochrane Handbook for the Systematic Reviews of Interventions^19^. Specifically, attention was focused on seven domains, i.e., random sequence generation, allocation concealment blinding of participants and personnel, blinding of the outcome assessments, incomplete outcome data, selective reporting and other biases. The review authors’ judgments were categorized as low risk, high risk, or unclear risk of bias. We categorized the risk of bias as unclear when no reported information could be used. If the investigators described a random component in the sequence generation process such as: a computer random number generator, shuffling envelopes or throwing dice, it was regarded as low risk. If odd or even date of birth, date of admission or hospital record number was used to randomise the patients, it was quasi-randomized and would be excluded in our article. If the participants and investigators enrolling participants could not foresee assignment when allocating the patients, it was regarded as low risk, and so forth. Any discrepancies were resolved by discussion within the review team.

### Statistical analysis

We present the results for the dichotomous data as summary risk ratios (RRs) and risk differences (RDs) with 95% the confidence intervals, and we report the weighted mean difference (WMD) for continuous data. When appropriate, the standardized mean difference (SMD) was also used to combine the trials that measured the same outcome via different methods.

The statistical analyses were performed with Stata (Version 12.0. StataCorp LLC, Lakeway Drive College Station, USA, http://www.stata.com/) and the risks of bias were recorded with RevMan software (Version 5.3. Copenhagen: The Nordic Cochrane Centre, The Cochrane Collaboration, 2014, http://community.cochrane.org/tools/review-production-tools/). Heterogeneity in the included studies was evaluated using the I2 statistic. If there was a substantial heterogeneity (I2 ≥ 50%), the trials were considered to be heterogeneous, and a different effects model or sensitivity analysis was selected to explore the source of the heterogeneity.

To evaluate the effects of PCD with or without other factors, we decided to perform the following subgroup analyses: non-instrumental delivery versus instrumental delivery, the use of uterotonics versus the non-use of uterotonics (or use after the delivery of the placenta), women with CCT versus those without CCT, and multigravida versus primigravida women.

We planned to use all of the primary outcomes in the subgroup analyses. If only a limited number of studies were available for a particular outcome, we evaluated the differences between the subgroups by inspecting the subgroups’ confidence intervals. Non-overlapping CIs were defined as statistically significant differences between the subgroups. If there were no studies available for a particular outcome, the comparison was not performed.

## Results

### Literature Search

**Figure 1:**
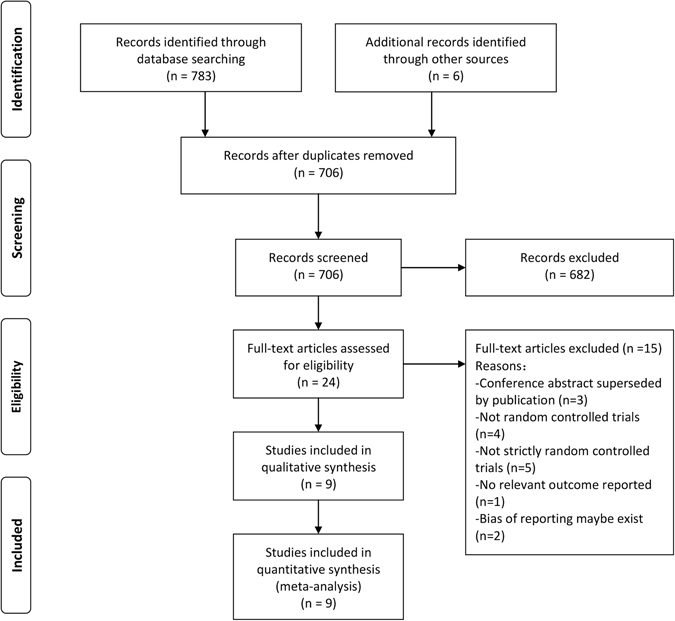
Article retrieval and screening.

We identified 789 articles in total, and 706 articles remained after the removal of the duplicates. After screening the titles and abstracts, we obtained 24 articles for full text review. During the title and abstract screening, studies were excluded mainly because they were not relevant. After full text review, 9 studies that compared PCD with non- cord drainage in the third stage of labour were included in the quantitative synthesis and meta-analysis (Fig. 1). Fifteen articles were excluded for the reasons illustrated in Fig. 1, and two of these studies was excluded based on the absence of the reporting of some outcomes of interest (The authors did not report the amount of postpartum haemorrhage in the standard way like other outcomes and failed to clarify the confusion between cord drainage and late-clamping in the article^13^. In another article PPH was not only defined as blood loss of more than 500 ml, but also a drop in haemoglobin of 3 gm/dl or more between admission and post delivery assessment which was confusing. The authors regarded blood loss as an outcome but failed to report it in the results^20^).

### Study Characteristics

**Figure 2:**
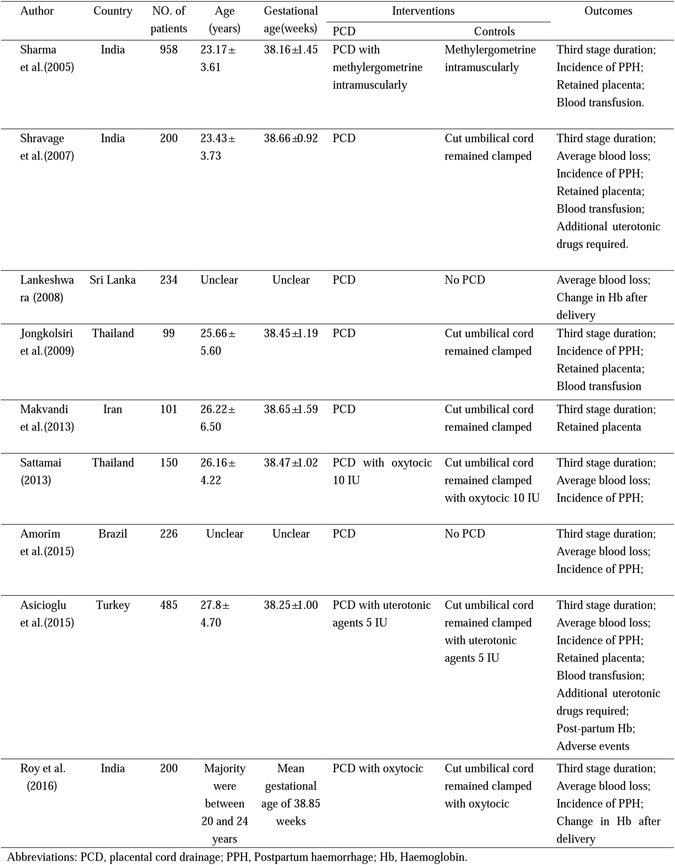
Characteristics of the trials identified and included in the quantitative synthesis.

Ultimately, 9 studies with 2653 participants were included in our analysis. These studies were published between 2005 and 2016. The primary characteristics of the studies are presented in Fig. 2. The mean ages of the women in the studies ranged from 20 years to 28 years. The mean gestational ages of the women were between 38 and 39 weeks. In all of the studies, the umbilical cord was clamped and cut immediately after vaginal birth in both groups. In the experimental groups, the umbilical cord was unclamped and drained, whereas in the control groups, the cut umbilical cord remained clamped. Uterotonics were used in 4 studies to manage the third stage of labour in both groups^11, 15, 16, 21^. In one study, a uterotonic was used after the expulsion of the placenta^12^. In 6 studies, instrumental deliveries were excluded^12, 15, 16, 18, 21, 22^. Sharma reported the outcomes separately for primigravida and multigravida women^11^. The women in Makvandi’s study were entirely primigravida^22^. We added these two studies to the primigravida subgroup.

With the exception of two studies that did not report detailed information about the method^17, 18^, all of the placentas included in 7 studies were delivered via CCT or the Brandt–Andrews maneuver when there were signs of placental separation. The duration of the third stage of labour was calculated as the time between the delivery of the neonate and the expulsion of the placenta. The blood from draining was contained in vessels such that the blood loss could be separately measured. However, the methods used to estimate postpartum blood loss varied. In 4 studies, blood loss in the third stage was measured by collecting the blood in certain containers^12, 16, 21, 23^, whereas Asicioglu *et al*. used weighing method to calculate the volume of blood loss^15^. There is no information about estimating blood loss in the other articles^11, 17, 18, 22^. In these studies, the retained placentas were left in place until the duration of the third stage reached 30 minutes, and the placentas were subsequently delivered by manual removal. PPH was defined as a loss of more than 500 mL of blood within the first 24 hours following childbirth.

The risk of bias is summarized in Supplementary Appendix S3. Except two studies that did not clarify rational random sequence generation (Amorim’s trial in 2015 and the study in 2008), all the included studies had low risk of bias in random sequence generation and reporting bias. Three of the included trials reported adequate concealment of allocations prior to assignment^15, 21, 23^. Only the study of Asicioglu reported blinding method and appeared to be of an adequate quality with a low risk of bias^15^. All data used in the meta-analysis are displayed in the Supplementary Table S1.

### Meta-analysis

**Figure 3:**
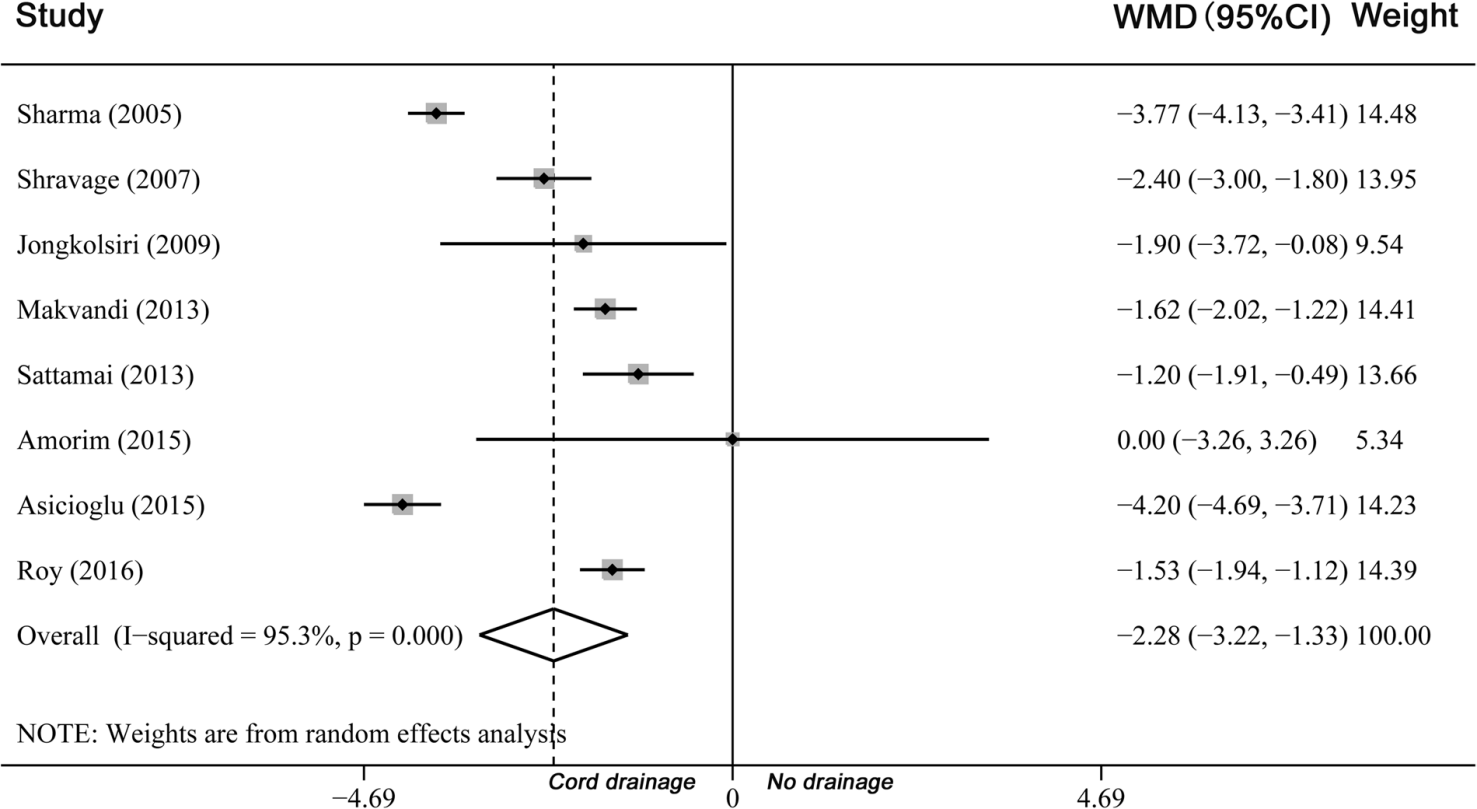
Comparison of cord drainage versus no drainage (all). Outcome: Length of the third stage of labour.

**Figure 4:**
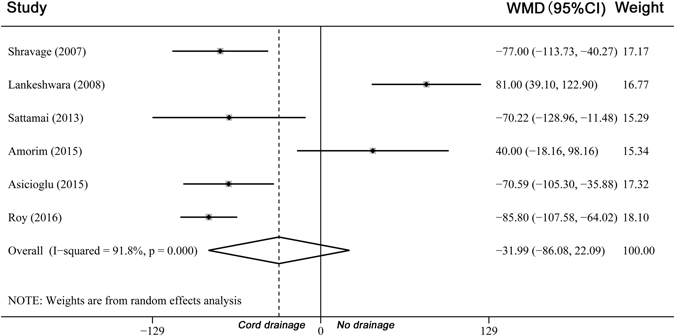
Comparison of cord drainage versus no drainage (all). Outcome: Average blood loss.

In a pooled analysis of all 9 trials, the length of third stage of labour was shorter among the groups of women who underwent cord drainage (WMD −2.28 minutes, 95% CI −3.22 to −1.33, heterogeneity: I² = 95.3%, Fig. 3). There was no significant difference in the amount of blood loss between groups (WMD −31.99 mL, 95% CI −86.08 to 22.09, heterogeneity: I² = 91.8%, Fig. 4). The incidence of PPH was less in the drainage groups compared with the control groups (RR 0.47, 95% CI 0.24 to 0.92, heterogeneity: I² = 54.0% and RD −0.03, 95% CI −0.06 to −0.01, heterogeneity: I² = 49.0%, Supplementary Figure S1).

Only one case of a retained placenta or the manual removal of the placenta was reported in the control groups of the included studies; thus, it was not possible to evaluate this factor (Supplementary Figure S2). There was no significant difference in the need for blood transfusions between the drainage and control groups (RR 0.53, 95% CI 0.23 to 1.22 and RD −0.01, 95% CI −0.03 to 0.01, Supplementary Figure S3). There was no difference in the prepartum haemoglobin levels between the groups (WMD 0.1 mg/dl, 95% CI −0.29 to 0.49), but the postpartum haemoglobin was higher in the cord drainage groups (WMD 0.75 mg/dl, 95% CI 0.62 to 0.87, Supplementary Figure S4). There was no difference in the changes in maternal haemoglobin after delivery between the groups (WMD −0.10 mg/dl, 95% CI −0.89 to 0.68, Supplementary Figure S5). Fewer women in the drainage groups required additional uterotonic drugs (RR 0.35, 95% CI 0.16 to 0.77 and RD −0.04, 95% CI, −0.07 to −0.01, Supplementary Figure S6). Adverse effects at the time of PCD were regarded as outcomes in only one study, and no adverse events were reported among any of the 262 women.

The results in Supplementary Figure S7 revealed that the mean differences were minimally altered in analyses that used fixed effect model. However when we used fixed effect model to analyse the amount of blood loss, the confidence intervals were narrower with significant difference between the drainage and control groups (WMD −53.56 mL, 95% CI −67.94 to −39.18, heterogeneity: I² = 91.8%, Supplementary Figure S8). We performed sensitivity analyses excluding two studies that did not clarify rational random sequence generation(Amorim’s trial in 2015 and the study in 2008). The great heterogeneity still exists in the analysis of the length of the third stage with the mean difference little changed (excluding Amorim’s trial due to missing data about the third stage duration in the study in 2008, Supplementary Figure S9), so does another sensitivity analysis excluding the study with outliers (the length of the third stage in Amorim’s trial was much longer than that of others, Supplementary Figure S9). However in another sensitivity analysis of blood loss, the heterogeneity disappeared after removing foregoing two studies (WMD −79.84 mL, 95% CI −95.71 to −63.97, heterogeneity: I² = 0%, Supplementary Figure S10). In addition, we examined the influence of each study on the overall estimate by excluding one study at a time and rerunning the meta-analysis in the analysis of the length of the third stage. Similar heterogeneity could be seen in the meta-analysis and the pooled estimate for the length of the third stage was nearly identical.

### Subgroup analyses

Three comparisons between subgroups were performed, but a comparison of the women who were managed with CCT and the women who did not receive CCT was not performed because CCT was applied to all of the participants.

The results of the subgroup analysis according to the mode of birth are displayed in Supplementary Table S2. The overlapping CIs for the subgroup analyses revealed that there were no differences between the subgroups in most of the outcomes. However, in the subgroup analysis of the incidence of PPH, the pre-existing substantial heterogeneity disappeared, and a reduction in the incidence of PPH was only observed among women in the drainage groups who experienced normal vaginal deliveries.

There were no significant differences in any of the outcomes among the subgroups that were defined according to the use of uterotonics or pregnancy history (Supplementary Tables S3 and S4). A reduction in the incidence of PPH was observed among the studies which did not limit their participants to the primigravida (Supplementary Table S4). For the other outcomes regarding the subgroups, the data were insufficient for analysis.

## Discussion

Our results demonstrate that PCD in the third stage of labour can shorten the third-stage duration by 2.28 minutes, but it can not significantly reduce the amount of blood loss when compared with clamping the umbilical cord. For women who experience normal vaginal deliveries, the incidence of PPH was reduced by 3%. Additionally, PCD could reduce the drop in haemoglobin during labour and thus lead to a higher level of postpartum haemoglobin. Finally, it could reduce the need for additional uterotonic drugs, and no adverse events were reported.

In our results, there is great heterogeneity in some meta-analyses. We used sensitivity analyses and subgroup analyses to explore the source of the heterogeneity. First, the potential explanation for the great heterogeneity in the analysis of the incidence of PPH may be the inclusion of assisted vaginal birth in some studies. It is obvious that assisted vaginal birth will increase the incidence of PPH^24, 25^. Second, in the analysis of blood loss, we found that Amorim’s trial in 2015 and the study in 2008 might be the source of the heterogeneity though it is difficult to explain due to the insufficient data about the studies, so random effect model was used to combine these studies. Finally in the sensitivity analyses of the third-stage duration, the mean differences were slightly altered with narrower confidence intervals when using fixed effect model although the high level of statistical heterogeneity would suggest that the random effects model used in the main analysis is a more suitable model. A meta-regression analysis was not performed due to the limited number of studies.

Our data do partially support the findings of an earlier Cochrane review that concluded that there are small reductions in the duration of the third stage of labour and in the amount of blood loss when cord drainage is used^14^. However, In the Cochrane review, only three trials with substantial consistency were included. Some of the results could not be compared due to the limited number of studies included. Given that a study was missed^17^ and recent data from additional studies were not included^15, 16, 18, 21, 22^, a review of this topic at this time is warranted. We found the amount of blood loss was not reduced when performing PCD, and we observed a reduction in the incidence of PPH during normal vaginal delivery and a higher level of postpartum haemoglobin among all women with vaginal deliveries.

Although women with normal vaginal deliveries may benefit from PCD, it is not really clinically significant. Whether the performance of this technique for such a small benefit is warranted remains inconclusive. However, PCD is simple, non-invasive and does not result in adverse events; thus, it may be easy to popularize. Additionally, PCD can be performed in all patients at any time. These advantages lend support to the use of PCD as a form of active management during the third stage of labour.

In 2012, the World Health Organization recommended that the cord be clamped between one and three minutes after birth unless the baby is asphyxiated and requires resuscitation^26^. Clearly, delayed clamping of the umbilical cord cannot reduce the incidence of PPH^27^. When umbilical cord clamping is delayed, it seems that the blood flow between the placenta and the baby through the umbilical cord continues^27^, and the process of placental transfusion is, in some aspects, similar to that of PCD. However, the amount of blood returned to the infant varies with the time of clamping and the level at which the infant is held (i.e., above or below the mother’s abdomen) before the clamping of the cord^28^. Therefore, delayed cord clamping may reduce the volume of blood that remains in the placenta, but it seems likely that there will still be some blood left in the placenta. For this reason, PCD can also be performed after delayed cord clamping.

In developed countries, blood loss can be reduced by various methods that include drugs, reproductive health care and more advanced obstetric care. However, the percentage of all maternal deaths that occurred among the bottom two quintiles of the socio-demographic index, in which haemorrhage is the dominant cause of maternal death, increased from approximately 68% in 1990 to more than 80% in 2015^1^. Challenges to improving reproductive health lie ahead in these countries. As a simple and free procedure, PCD is quite appropriate for women in these countries. PCD can be performed anywhere, even at home, and it has no adverse effects. We suggest that PCD should be evaluated in revised guidelines aiming to improve revision for improvements in maternal health.

PCD was performed immediately after the clamping and cutting of the cord in all of the studies. It will be interesting to evaluate the effects of cord drainage in relation to the timing of the commencement of CCT. When cord drainage is performed with CCT or the Brandt–Andrews maneuver, no extra work is needed in the third stage of labour, which makes cord drainage more convenient.

The limitations of this analysis are clear. First, only 9 studies were included in the analysis, an exploration for publication bias was not performed, however we could not deny the possibility of un-published studies, especially those with negative results, and this issue might have affected the accuracy of the results. Second, detailed information and data regarding 460 women in Amorim’s trial in 2015 and the study in 2008, such as the incidence of postpartum haemorrhage, the length of the third stage of labour, were lacking, and this lack of information might be a source of deviation. Finally, among the included participants, only one retained placenta was reported. No adverse events were reported among the 262 women during the drainage period. The relative rarity of adverse events may indicate the need for a larger study populations to enable a meaningful comparison. However, we have introduced a topic of clinical importance, i.e., cord drainage, which is a simple procedure that can potentially be performed in all births. All of the studies included in our analysis had appropriate sample sizes, and the risks of bias were acceptable.

The physiological process of PCD has not been clear and thus requires further research. Future studies should also focus on the effects of and adverse events associated with cord drainage in different circumstances.

In conclusion, placental cord drainage during the third stage of labour can shorten the third-stage duration and reduce the incidence of postpartum haemorrhage among women with normal vaginal deliveries. Cord drainage is a simple and non-invasive procedure that should be considered after delayed cord clamping. Further studies regarding the physiological processes and effects of placental cord drainage in additional circumstances are needed.

## Electronic supplementary material

Supplementary information
