# Supplementary material for: Effects of placental cord drainage in the third stage of labour: A meta-analysis
Source: Sci Rep. 2017 Aug 1;7:7067. doi: 10.1038/s41598-017-07722-7 (PMC5539148; doi:10.1038/s41598-017-07722-7)
Supplement: Supplementary file 1 — Supplementary information [file 41598_2017_7722_MOESM1_ESM.pdf]

Effects of placental cord drainage in the third stage of labour: A meta-analysis

Hang-lin Wu[1*], Xiao-wen Chen[1], Pei Wang[1], Qiu-meng Wang[1]

[1] Department of Obstetrics and Gynaecology, Hangzhou Women's Hospital, Hangzhou, Zhejiang, China. Correspondence and requests for materials should be addressed to H.L.W. (email: hanglinwu@gmail.com)

Supplementary Appendix S2. Detailed search strategy for the meta-analysis

Databases searched	PubMed, Embase, the Cochrane Library, Web of Science and Google Scholar.
Search strategy for Pubmed	(((((((cord) OR placenta*) AND drain*)) OR Drainage[MeSH Terms])) AND ((((((Stage) OR Stages) AND (((third) OR 3) OR 3rd))) OR ((((((((((((((Postpartum Hemorrhage[MeSH Terms]) OR Hemorrhage, Postpartum) OR Immediate Postpartum Hemorrhage) OR Hemorrhage, Immediate Postpartum) OR Postpartum Hemorrhage, Immediate) OR Delayed Postpartum Hemorrhage) OR Hemorrhage, Delayed Postpartum) OR Postpartum Hemorrhage, Delayed)) OR post-partum haemorrhage) OR postpartum haemorrhage) OR post-partum hemorrhage)))) OR (("Placenta, Retained/prevention and control"[Mesh])))) AND (("randomized controlled trial"[pt] OR "controlled clinical trial"[pt] OR randomized[tiab] OR placebo[tiab] OR "drug therapy"[sh] OR randomly[tiab] OR trial[tiab] OR groups[tiab])).
Other sources	50 related journals were also searched, the majority of which are currently on the initiative lists of Core Outcomes in Women's and Newborn Health. The reference lists of the selected articles and reviews were hand searched to identify any other relevant articles.

Journals searched are the following:
1.Acta Obstetricia et Gynecologica Scandinavica
2.American Journal of Obstetrics & Gynecology
3. American Journal of Perinatology
4. Australian and New Zealand Journal of Obstetrics and Gynaecology
5. Best Practice & Research: Clinical Obstetrics & Gynaecology
6. Birth: Issues in Perinatal Care
7. BJOG: An International Journal of Obstetrics and Gynaecology
8. BMC Pregnancy and Childbirth
9. BMC Women's Health
10. Clinical Obstetrics and Gynecology
11. Clinics in Perinatology
12. Cochrane Gynaecology and Fertility Group
13. Cochrane Fertility Regulation Group
14. Cochrane Pregnancy and Childbirth Group
15. Contraception
16.Current Obstetrics and Gynecology Reports
17.Current Opinion in Obstetrics and Gynecology
18. European Journal of Obstetrics & Gynecology and Reproductive Biology
19. Fertility and Sterility

20. Fertility Research and Practice
21. Female Pelvic Medicine and Reconstructive Surgery
22. Fetal Diagnosis and Therapy
23. Geburtshilfe & Frauenheikunde
24. Ginekologia Polska
25. Gynecological Surgery
26. Gynecologic and Obstetric Investigation
27. Gynecologic Oncology
28. Human Fertility
29. Human Reproduction
30. International Journal of Gynecology & Obstetrics
31. Journal de Gynecologie Obstétrique et Biologie de la Reproduction
32. Journal of Obstetrics and Gynecology of India
33. Journal of Midwifery & Women's Health
34. Journal of Obstetrics & Gynaecology
35. Journal of Obstetrics and Gynaecology Canada
36. Journal of Obstetrics and Gynaecology Research
37. Journal of Obstetric, Gynecologic & Neonatal Nursing
38. Maternal Health, Neonatology and Perinatology
39. Midwifery
40. Nederlands Tijdschrift voor Obstetrie en Gynaecologie (NTOG)
41. New Zealand College of Midwives Journal
42. Obstetrics & Gynecology
43. Placenta
44.Post Reproductive Health
45. Reproductive Health
46. Russian Journal of Obstetrics and Gynaecology
47.Shiraz E Medical Journal
48. Women and Birth
49.Women's Midlife Health
50. Medical Journal of Srisaket Surin Buriram Hospitals

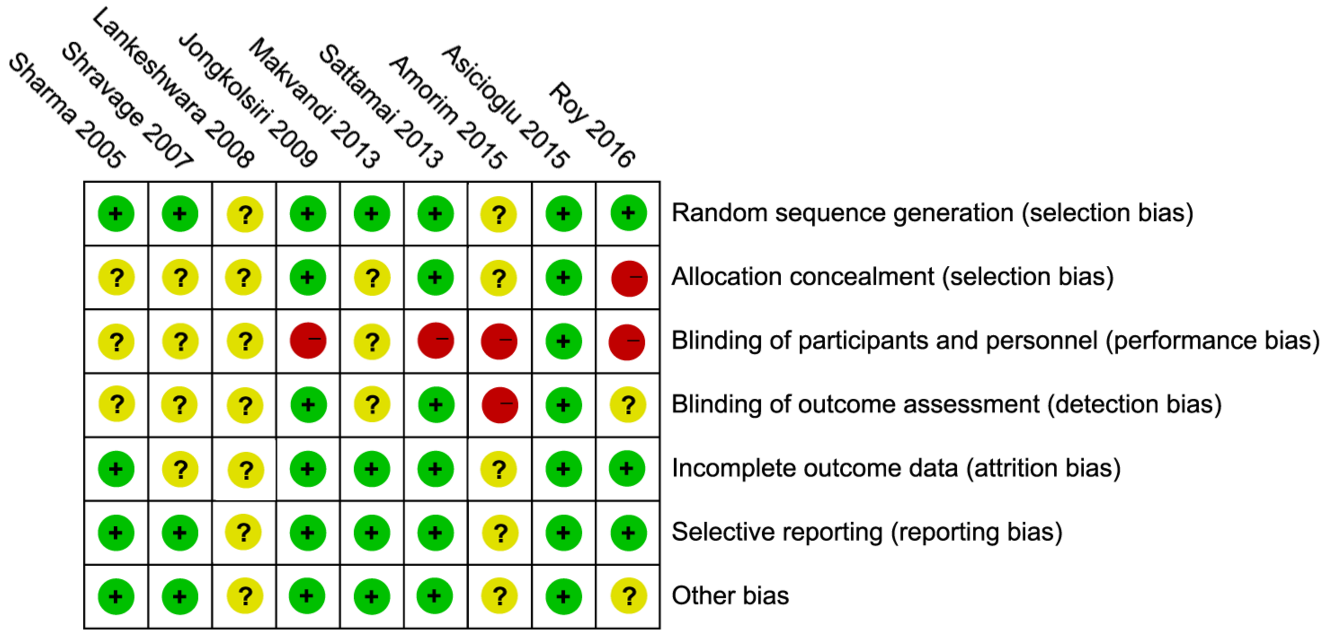

Supplementary Appendix S3. Risks of bias of included studies. The plus sign indicates a low risk of bias; the minus sign indicates a high risk of bias; the question mark indicates an unclear risk of bias.

Supplementary Table S1. All data underlying the findings in the manuscript

Third stage duration(min)							
Study	Year	Tsample	Tmean	Tsd	Csample	Cmean	Csd
Sharma	2005	478	3.22	2.82	480	6.99	2.86
Shravage	2007	100	5.02	1.71	100	7.42	2.56
Jongkolsiri	2009	49	5.1	2.4	50	7	6.1
Makvandi	2013	50	3.54	0.91	51	5.16	1.13
Sattamai	2013	50	2.4	1.8	50	3.6	1.8
Amorim	2015	113	14	13	113	14	12
Asicioglu	2015	242	3.5	1.9	243	7.7	3.4
Roy	2016	100	3.51	1.39	100	5.04	1.57

Average blood loss(ml)							
Study	Year	Tsample	Tmean	Tsd	Csample	Cmean	Csd
Shravage	2007	100	175.05	118.15	100	252.05	145.48
Lankeshwara	2008	117	222	208	117	141	101
Sattamai	2013	50	261.18	111.41	50	331.4	180.29
Amorim	2015	113	248	254	113	208	187
Asicioglu	2015	242	207.04	123.3	243	277.63	246.9
Roy	2016	100	227.5	75.3	100	313.3	81.7

Incidence of Postpartum hemorrhage							
Study	Year	Tevent	Tnoevent	Tsample	Cevent	Cnoevent	Csample
Sharma	2005	38	440	478	41	439	480
Shravage	2007	3	97	100	10	90	100
Jongkolsiri	2009	0	49	49	0	50	50
Sattamai	2013	2	48	50	7	43	50
Asicioglu	2015	8	234	242	16	227	243
Roy	2016	1	99	100	9	91	100

Retained placenta or manual removal of placenta							
Study	Year	Tevent	Tnoevent	Tsample	Cevent	Cnoevent	Csample
Sharma	2005	0	478	478	0	480	480
Shravage	2007	0	100	100	0	100	100
Jongkolsiri	2009	0	49	49	1	49	50
Makvandi	2013	0	50	50	0	51	51
Asicioglu	2015	0	242	242	0	243	243

Blood transfusion							
Study	Year	Tevent	Tnoevent	Tsample	Cevent	Cnoevent	Csample
Sharma	2005	7	471	478	9	471	480
Jongkolsiri	2009	0	49	49	0	50	50
Asicioglu	2015	4	238	242	12	231	243

Change of maternal hemoglobin after delivery(mg/dl)

Study	Year	Tsample	Tmean	Tsd	Csample	Cmean	Csd
Lankeshwara	2008	117	1.3	0.7	117	1	0.6
Roy	2016	100	0.6	0.3	100	1.1	0.25

Pre-partum maternal hemoglobin(mg/dl)

Study	Year	Tsample	Tmean	Tsd	Csample	Cmean	Csd
Asicioglu	2015	242	10.9	0.9	243	11	1.1
Roy	2016	100	10.2	0.56	100	9.9	0.58

Postpartum maternal hemoglobin(mg/dl)

Study	Year	Tsample	Tmean	Tsd	Csample	Cmean	Csd
Asicioglu	2015	242	9.8	1	243	9.1	0.9
Roy	2016	100	9.6	0.62	100	8.8	0.71

Additional uterotonic drugs required

Study	Year	Tevent	Tnoevent	Tsample	Cevent	Cnoevent	Csample
Shravage	2007	3	97	100	7	93	100
Asicioglu	2015	5	237	242	16	227	243

Adverse events at time of drainage

Study	Year	Tevent	Tnoevent	Tsample	Cevent	Cnoevent	Csample
Asicioglu	2015	0	242	242	0	243	243

Abbreviations: Tsample, Sample size of treatment group; Csample, Sample size of control group; Tevent, Number of events happened in treatment group; Tnoevent, Number of samples without event occurred in treatment group; Cevent, Number of events happened in control group; Cnoevent, Number of samples without event occurred in control group; Tmean, Mean of results in treatment group; Tsd, Standard deviation of results in treatment group; Cmean, Mean of results in control group; Csd, Standard deviation of results in control group.

Supplementary Figure S1 . Comparison of cord drainage versus no drainage (all)

Outcome:Incidence of postpartum haemorrhage(A:risk ratio,B:risk difference)

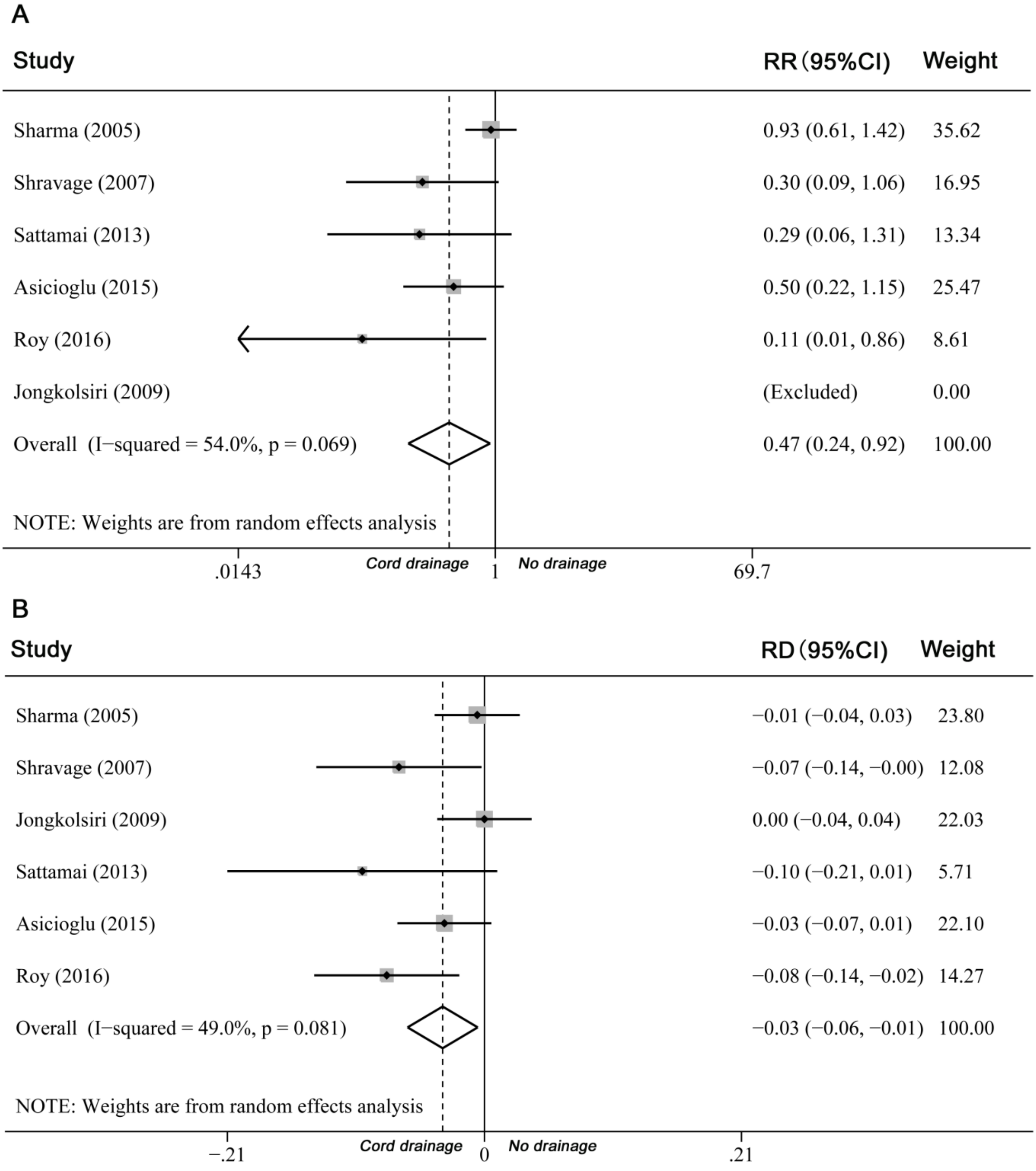

Supplementary Figure S2 . Comparison of cord drainage versus no drainage (all)

Outcome:Retained placenta or manual removal of placenta(A:risk ratio,B:risk difference)

A

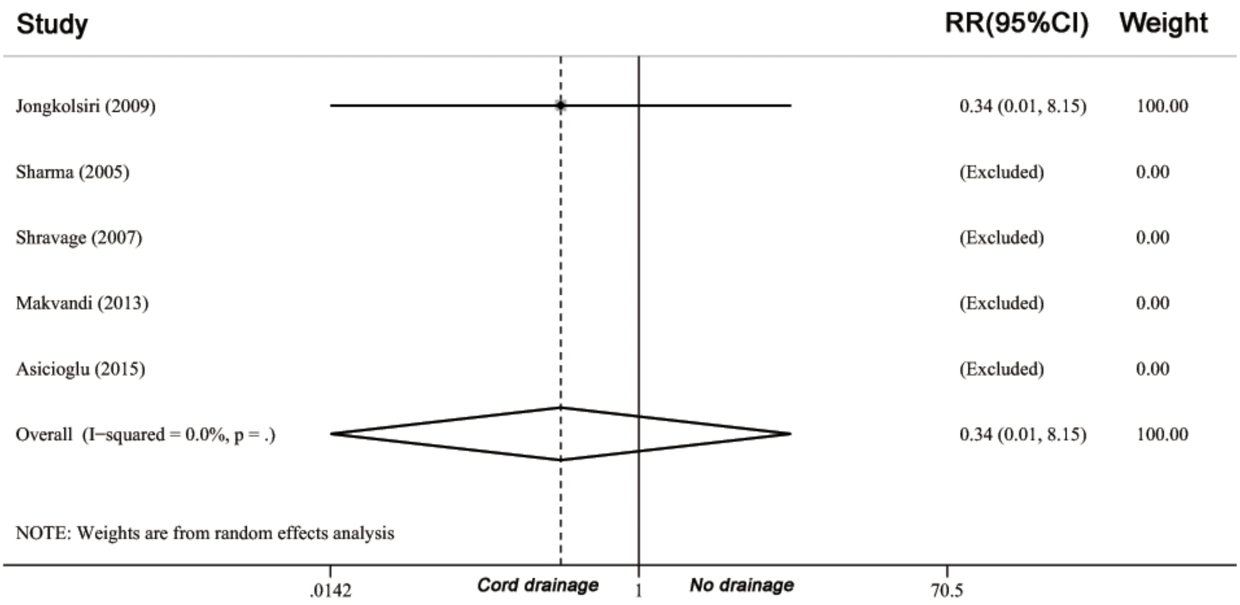

B

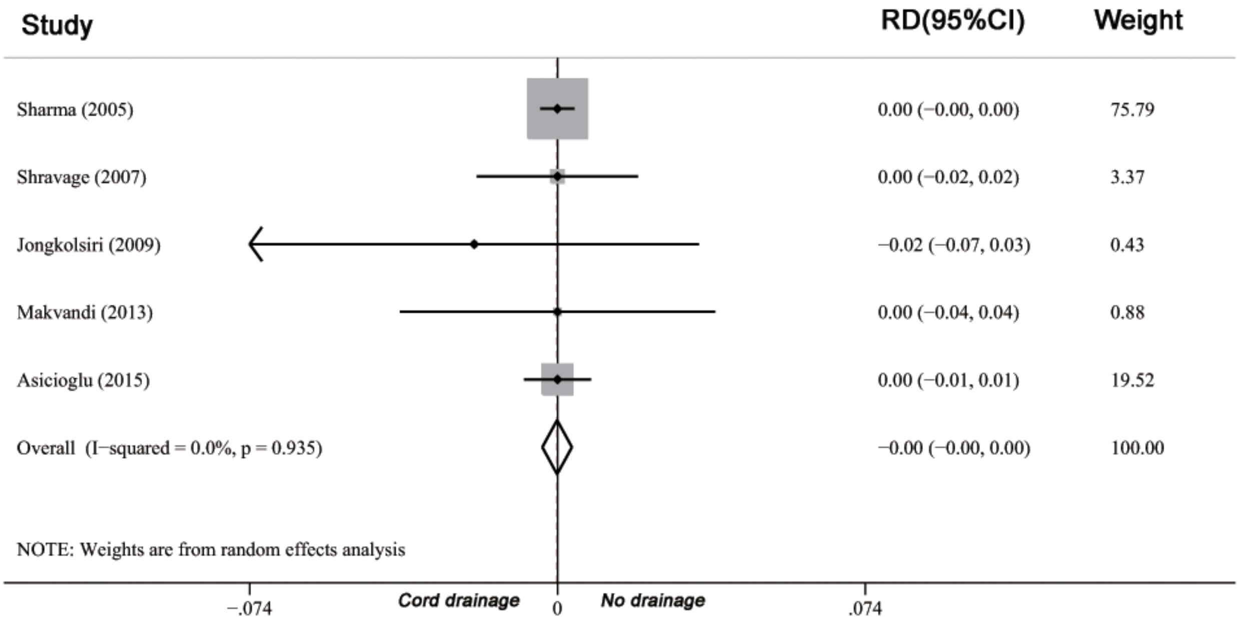

Supplementary Figure S3 . Comparison of cord drainage versus no drainage (all)

Outcome:Need for blood transfusion(A:risk ratio,B:risk difference)

A

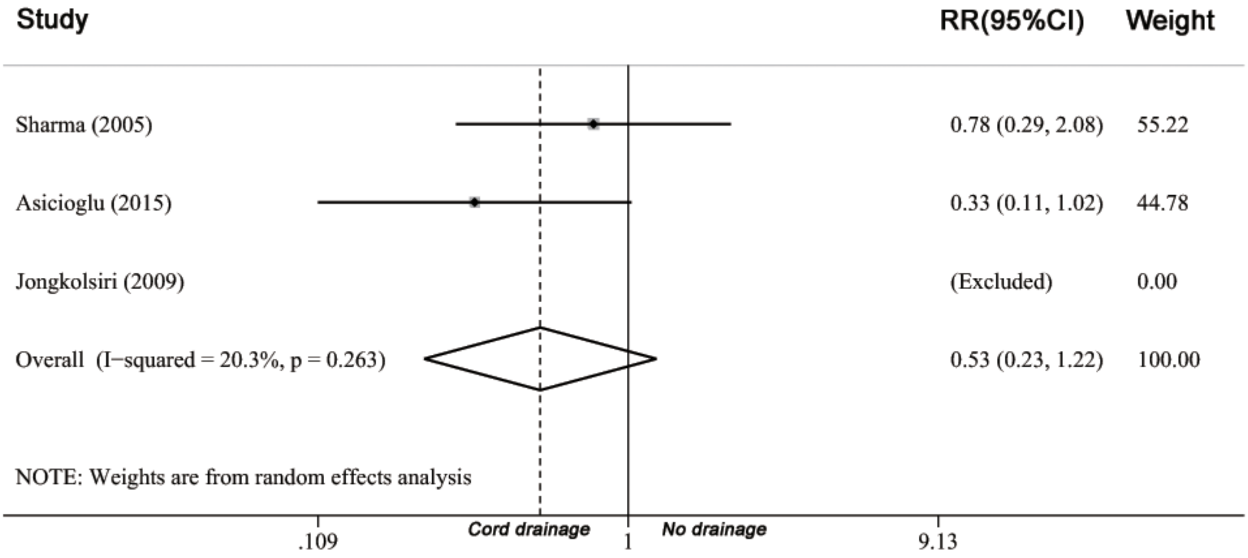

B

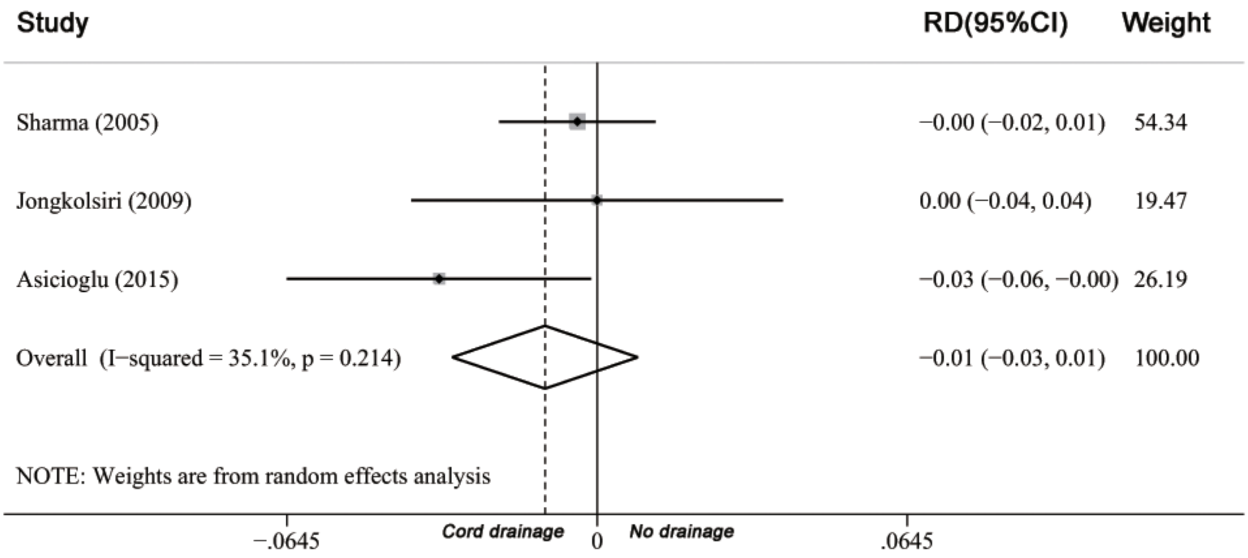

Supplementary Figure S4 . Comparison of cord drainage versus no drainage (all)

Outcome:prepartum hemoglobin(Hb) and postpartum hemoglobin(Hb)

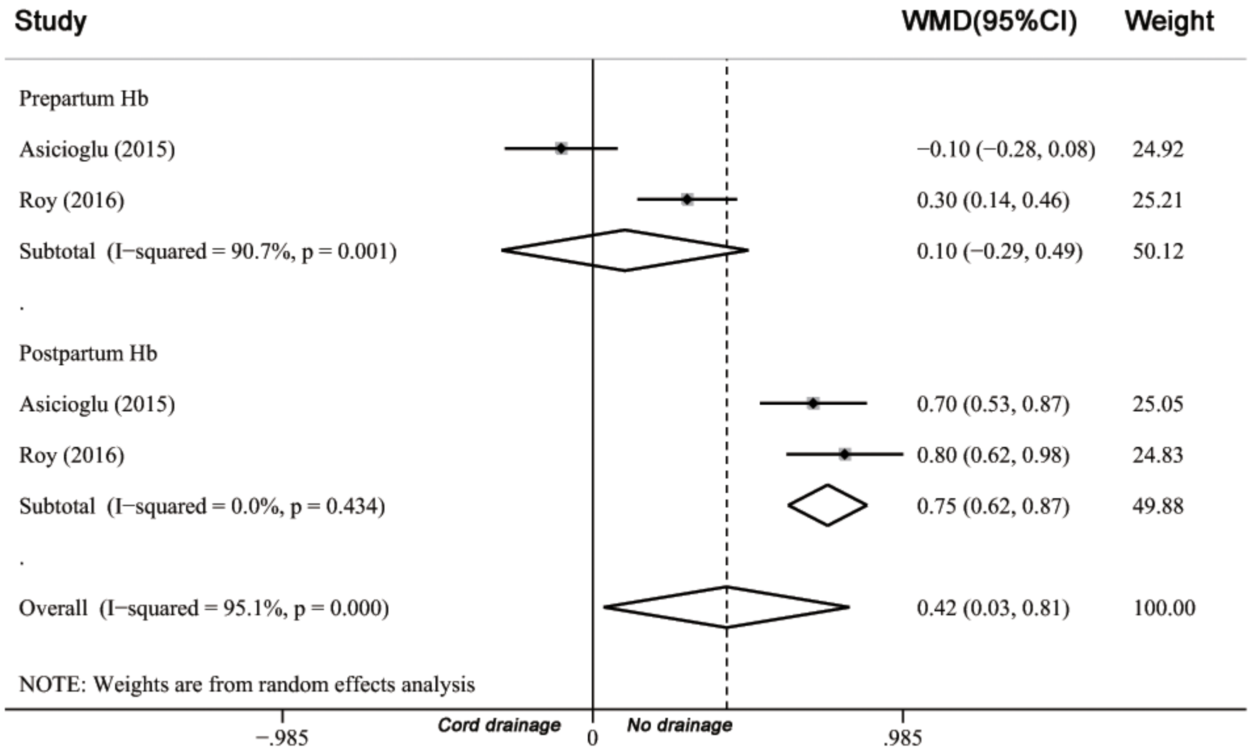

Supplementary Figure S5. Comparison of cord drainage versus no drainage (all)

Outcome:Changes in maternal haemoglobin after delivery

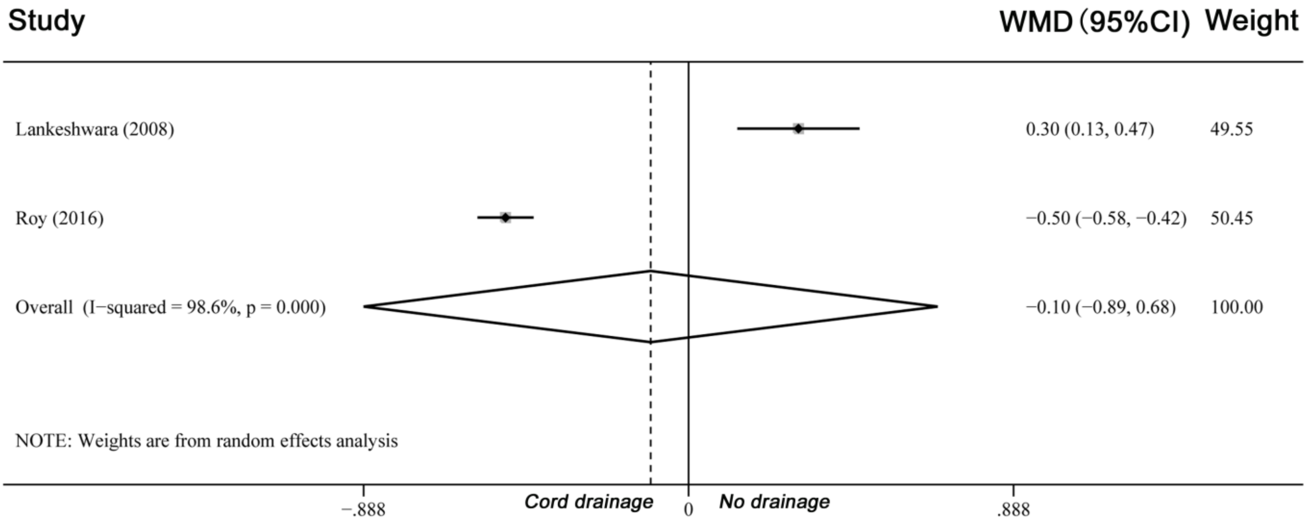

Supplementary Figure S6. Comparison of cord drainage versus no drainage (all)

Outcome:Additional uterotonic drugs required(A:risk ratio,B:risk difference)

A

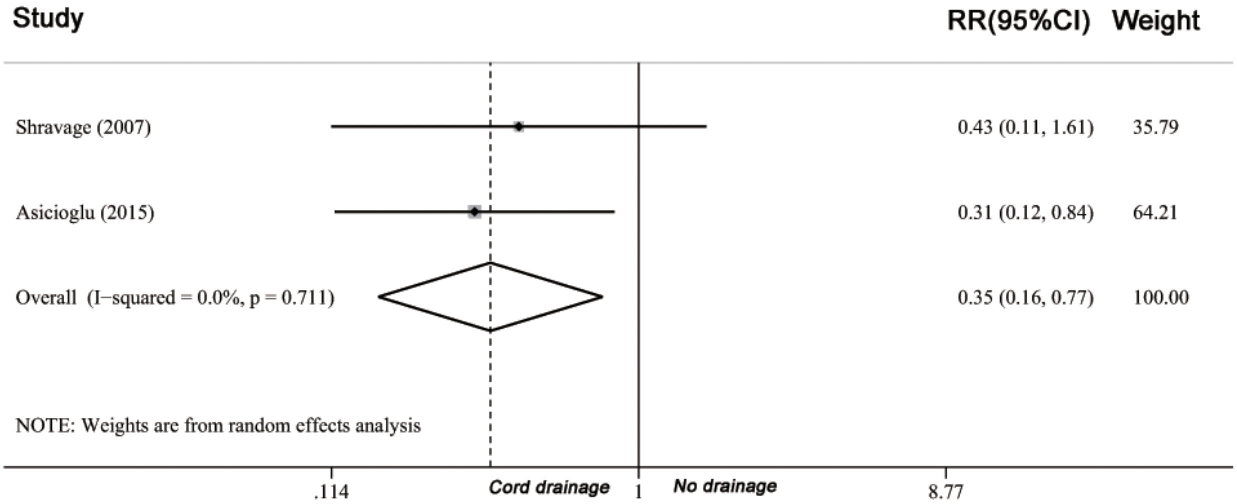

B

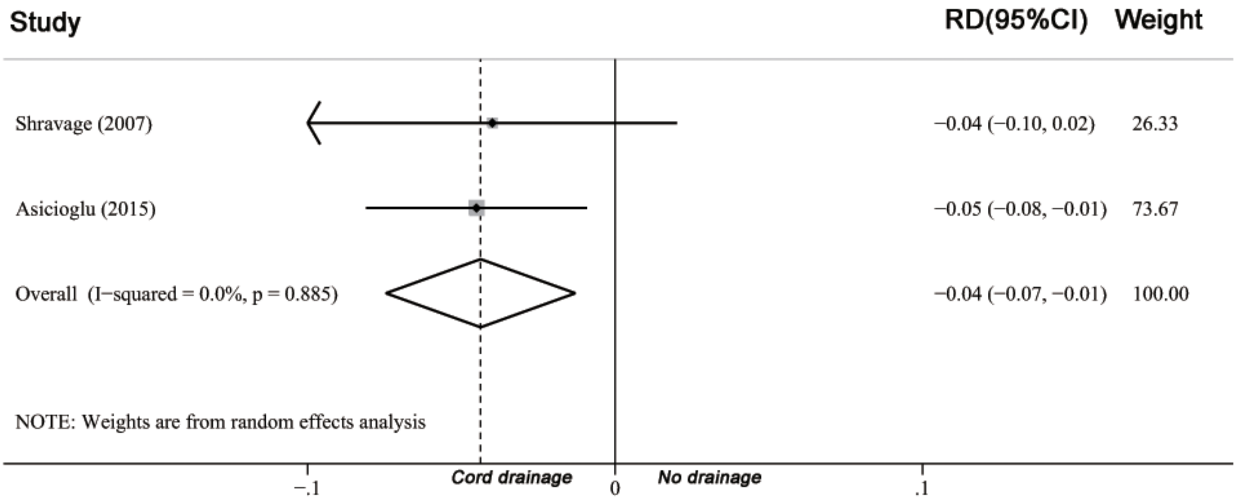

Supplementary Figure S7 . Comparison of cord drainage versus no drainage (all)

Outcome:Length of the third stage of labour

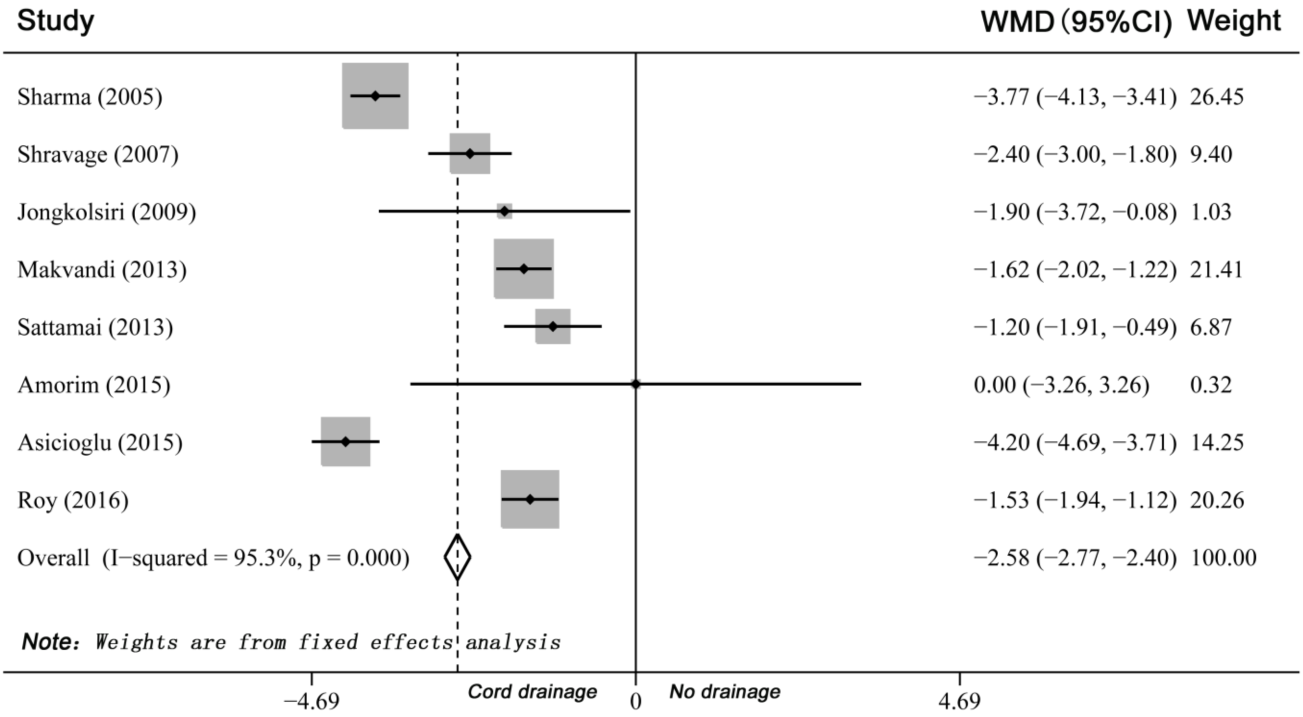

Supplementary Figure S8. Comparison of cord drainage versus no drainage (all)

Outcome:Average blood loss

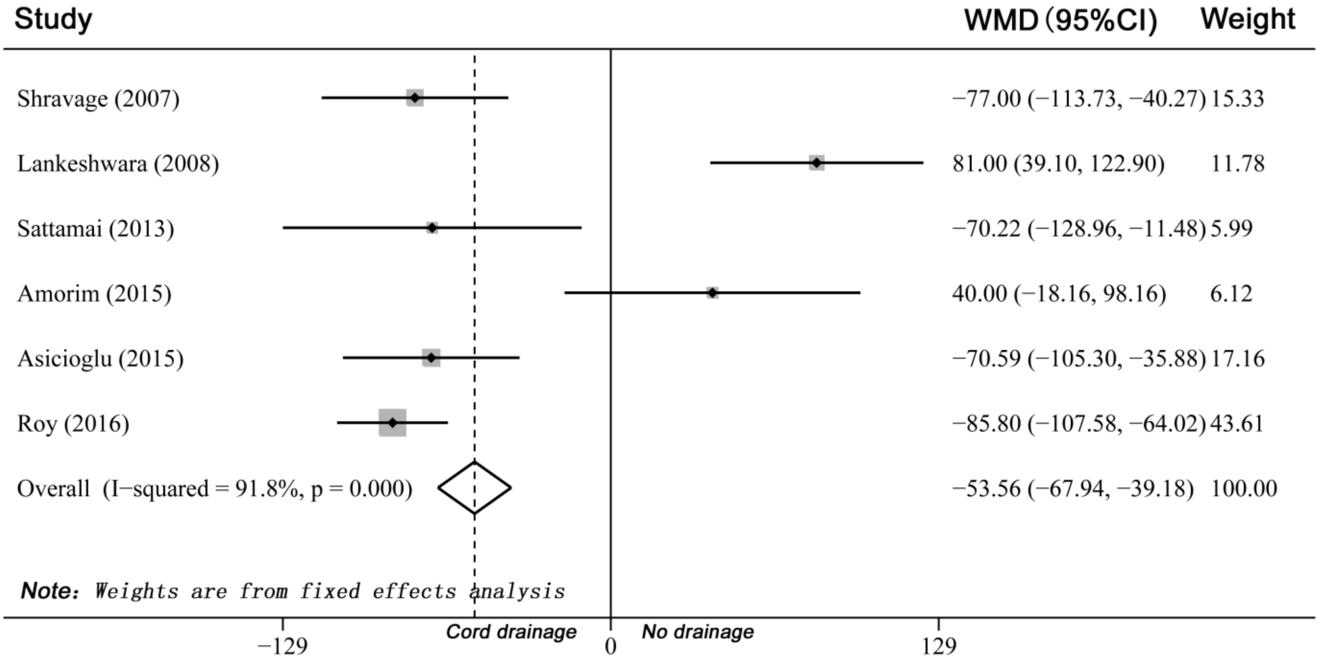

Supplementary Figure S9 . Comparison of cord drainage versus no drainage (*)

Outcome:Length of the third stage of labour

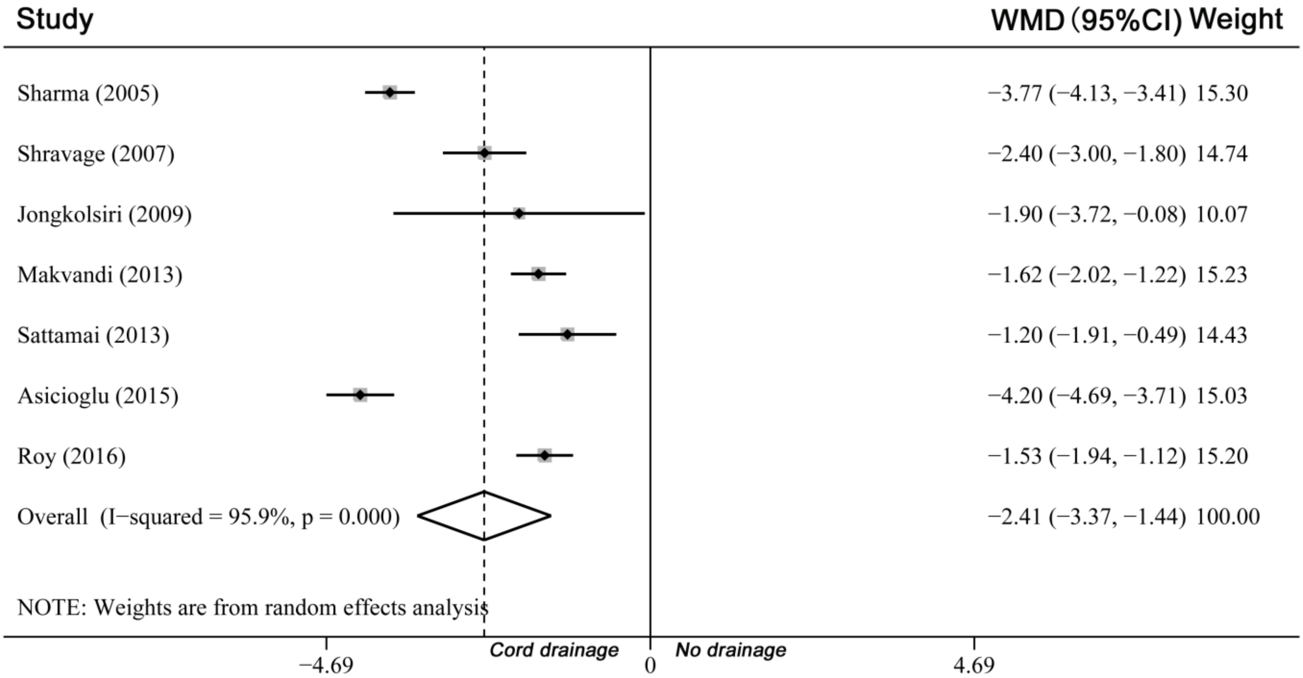

*Sensitivity analysis by excluding Amorim 2015 (The article did not clarify rational random sequence generation and reported the third stage duration with outliers)

Supplementary Figure S10 . Comparison of cord drainage versus no drainage (*)

Outcome:Average blood loss

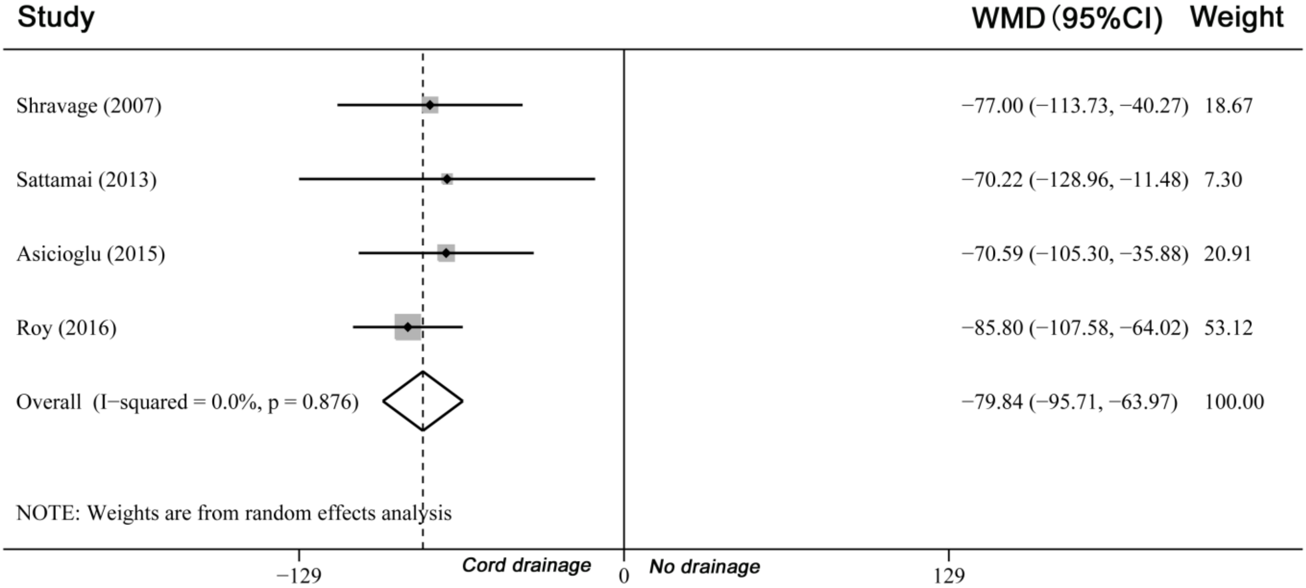

*Sensitivity analysis by excluding Amorim 2015 and Lankeshwara 2008 (The articles did not clarify rational random sequence generation)

Supplementary Table S2. Cord drainage versus no drainage: subgroups according to the mode of birth (normal vaginal birth versus mixed with assisted vaginal birth)

Outcome or subgroup title	*No. of studies*	*No. of participants*	*Statistical method*	*Effect size*	*Heterogeneity (I^2)*
**1.Third stage duration**					
•normal vaginal birth	6	1362	MD(IV, Random, 95% CI)	-2.05(-3.07,-1.03)	94.6%
•mixed with assisted vaginal birth	2	1057	MD (IV, Random, 95% CI)	-3.06(-4.84,-1.28)	74.4%
**2.Incidence of postpartum haemorrhage**					
•normal vaginal birth	4	1035	RR (M-H, Random, 95% CI)	0.36(0.20,0.65)	0.0%
			RD (M-H, Random, 95% CI)	-0.05(-0.08,-0.03)	0.0%
•mixed with assisted vaginal birth	2	1057	RR (M-H, Random, 95% CI)	0.93(0.61,1.42)	Not available
			RD (M-H, Random, 95% CI)	0.00(-0.03,0.02)	0.0%
**3.Retained placenta**					
•normal vaginal birth	3	786	RR (M-H, Random, 95% CI)	Excluded	Not available
			RD (M-H, Random, 95% CI)	0.00(-0.01,0.01)	0.0%
•mixed with assisted vaginal birth	2	1057	RR(M-H, Random, 95% CI)	0.34(0.01,8.15)	Not available
			RD (M-H, Random, 95% CI)	0.00(-0.02,0.02)	19.5%
**4.Blood transfusion**					
•normal vaginal birth	1	485	RR (M-H, Random, 95% CI)	0.34(0.11,1.02)	Not available
			RD (M-H, Random, 95% CI)	-0.03(-0.06,0.00)	Not available
•mixed with assisted vaginal birth	2	1057	RR(M-H, Random, 95% CI)	0.78(0.29,2.08)	Not available
			RD (M-H, Random, 95% CI)	0.00(-0.02,0.01)	0.0%

Abbreviations: MD, Mean Difference; RR, Risk Ratio; RD, Risk Difference; IV, Inverse Variance; M-H, Mantel-Haenszel.

Supplementary Table S3. Cord drainage versus no drainage: subgroups according to the use of uterotonics in the third of stage of labour (use of uterotonics versus non-use of uterotonics)

Outcome or subgroup title	*No. of studies*	*No. of participants*	*Statistical method*	*Effect size*	*Heterogeneity (I^2)*
**1.Third stage duration**					
•use of uterotonics	4	1793	MD (IV, Random, 95% CI)	-2.69 (-4.12, -1.25)	97.4%
•non-use of uterotonics	2	301	MD (IV, Random, 95% CI)	-1.98 (-2.74, -1.21)	77.6%
**2.Average blood loss**					
•use of uterotonics	3	835	MD (IV, Random, 95% CI)	-80.49(-98.09 ,-62.89)	0.0%
•non-use of uterotonics	1	200	MD (IV, Random, 95% CI)	-77.00(-113.73,-40.27)	Not available
**3.Incidence of postpartum haemorrhage**					
•use of uterotonics	4	1793	RR (M-H, Random, 95% CI)	0.51(0.24, 1.08)	56.7%
			RD (M-H, Random, 95% CI)	-0.04(-0.08,0.00)	51.7%
•non-use of uterotonics	1	200	RR (M-H, Random, 95% CI)	0.30(0.09, 1.06)	Not available
			RD (M-H, Random, 95% CI)	-0.07(-0.14,0.00)	Not available
**4.Retained placenta**					
•use of uterotonics	2	1443	RR (M-H, Random, 95% CI)	Not available	Not available
			RD (M-H, Random, 95% CI)	0.00(0.00,0.00)	0.0%
•non-use of uterotonics	2	301	RR (M-H, Random, 95% CI)	Not available	Not available
			RD (M-H, Random, 95% CI)	0.00(-0.02,0.02)	0.0%
**5.Additional uterotonic drugs needed**					
•use of uterotonics	1	485	RR (M-H, Random, 95% CI)	0.31(0.12, 0.84)	Not available
			RD (M-H, Random, 95% CI)	-0.05(-0.08,-0.01)	Not available
•non-use of uterotonics	1	200	RR (M-H, Random, 95% CI)	0.43(0.11, 1.61)	Not available
			RD (M-H, Random, 95% CI)	-0.04(-0.10,0.02)	Not available

Abbreviations: MD, Mean Difference; RR, Risk Ratio; RD, Risk Difference; IV, Inverse Variance; M-H, Mantel-Haenszel.

Supplementary Table S4. Cord drainage versus no drainage: subgroups according to parturition history(primigravida versus mixed with multigravida)

Outcome or subgroup title	*No. of studies*	*No. of participants*	*Statistical method*	*Effect size*	*Heterogeneity (I^2)*
**1.Third stage duration**					
•Primigravida	2	421	MD(IV, Random, 95% CI)	-2.70(-4.80, -0.59)	98.4%
•Mixed with multigravida	6	1360	MD(IV, Random, 95% CI)	-2.09(-3.29, -0.89)	93.9%
**2.Incidence of postpartum haemorrhage**					
•Primigravida	1	320	RR (M-H, Random, 95% CI)	0.93(0.61, 1.42)	Not available
			RD (M-H, Random, 95% CI)	-0.01(-0.04,0.03)	Not available
•Mixed with multigravida	5	1134	RR(M-H, Random, 95% CI)	0.36(0.20, 0.65)	0.0%
			RD (M-H, Random, 95% CI)	-0.05(-0.08,-0.01)	58.6%
**3.Retained placenta**					
•Primigravida	2	421	RR(M-H, Random, 95% CI)	Not available	Not available
			RD (M-H, Random, 95% CI)	0.00(0.00,0.00)	0.0%
•Mixed with multigravida	3	784	RR(M-H, Random, 95% CI)	0.34(0.01, 8.15)	Not available
			RD (M-H, Random, 95% CI)	0.00(-0.01,0.01)	0.0%
**4.Blood transfusion**					
•Primigravida	1	320	RR(M-H, Random, 95% CI)	0.78(0.29, 2.08)	Not available
			RD (M-H, Random, 95% CI)	0.00(-0.02,0.01)	Not available
•Mixed with multigravida	2	584	RR(M-H, Random, 95% CI)	0.33(0.11, 1.02)	Not available
			RD (M-H, Random, 95% CI)	-0.02(-0.05,0.02)	50.9%

Abbreviations: MD, Mean Difference; RR, Risk Ratio; RD, Risk Difference; IV, Inverse Variance; M-H, Mantel-Haenszel.
